# Alternagin-C (ALT-C), a Disintegrin-Like Cys-Rich Protein Isolated from the Venom of the Snake *Rhinocerophis alternatus*, Stimulates Angiogenesis and Antioxidant Defenses in the Liver of Freshwater Fish, *Hoplias malabaricus*

**DOI:** 10.3390/toxins9100307

**Published:** 2017-09-28

**Authors:** Diana Amaral Monteiro, Heloisa Sobreiro Selistre-de-Araújo, Driele Tavares, Marisa Narciso Fernandes, Ana Lúcia Kalinin, Francisco Tadeu Rantin

**Affiliations:** Department of Physiological Sciences, Federal University of São Carlos (UFSCar), São Carlos, SP 13565-905, Brazil; hsaraujo@ufscar.br (H.S.S.-d.-A.); driele.tavares@gmail.com (D.T.); dmnf@ufscar.br (M.N.F.); akalinin@ufscar.br (A.L.K.); ftrantin@ufscar.br (F.T.R.)

**Keywords:** disintegrin, blood vessel formation, VEGF, antioxidant enzymes, oxidative stress biomarkers

## Abstract

Alternagin-C (ALT-C) is a disintegrin-like protein isolated from *Rhinocerophis alternatus* snake venom, which induces endothelial cell proliferation and angiogenesis. The aim of this study was to evaluate the systemic effects of a single dose of alternagin-C (0.5 mg·kg^−1^, via intra-arterial) on oxidative stress biomarkers, histological alterations, vascular endothelial growth factor (VEGF) production, and the degree of vascularization in the liver of the freshwater fish traíra, *Hoplias malabaricus*, seven days after the initiation of therapy. ALT-C treatment increased VEGF levels and hepatic angiogenesis. ALT-C also enhanced hepatic antioxidant enzymes activities such as superoxide dismutase, catalase, glutathione peroxidase, and glutathione reductase, decreasing the basal oxidative damage to lipids and proteins in the fish liver. These results indicate that ALT-C improved hepatic tissue and may play a crucial role in tissue regeneration mechanisms.

## 1. Introduction

Snake venoms contain a complex pool of proteins (more than 90% of the dry weight), organic compounds with a low molecular mass, and inorganic compounds [1]. Among these compounds are acetylcholinesterases, ADPases, phospholipases, hialuronidases, and hemostasis active compounds such as metalloproteases, named the snake venom metalloproteases (SVMPs), and serinoproteases [2].

Disintegrins of snake venoms are mostly derived from proteolytically processed precursor forms having a metalloprotease (SVMP) domain [3]. Alternagin-C (ALT-C) is an ECD (Glu-Cys-Asp sequence)-containing disintegrin-like/cysteine-rich domain released from metalloprotease alternagin isolated from the crude venom of the snake *Rhinocerophis alternatus*, popularly known in South America as urutu. Disintegrin-like proteins trigger integrin-mediated intracellular signal transduction events that modify gene expression and cell responses and interfere with cell-cell and cell-matrix interactions in a bi-directional manner across cell membranes [4,5].

Previous studies demonstrated that ALT-C induces endothelial cell proliferation and angiogenesis both in vitro and in vivo by up-regulating the expression of vascular endothelial growth factor (VEGF) and its receptors [4,6]. ALT-C binds to *α*_2_*β*_1_ integrin, a major collagen receptor, competitively inhibiting cell adhesion to collagen, triggering downstream signaling molecules, and inducing a significant increase in several genes related to cell cycle control, including VEGF and other growth factors such as inducible early growth response, interleukin 11, early growth response 2 and 3, and the insulin-induced gene [7]. ALT-C also induced significant cytoskeleton dynamic changes with the polymerization of F-actin, focal adhesion kinase (FAK), and phosphoinositol 3-kinase (PI3K) activation, as well as erk-2 translocation [3].

An integrin-binding peptide, such as ALT-C, able to up-regulate the expression of growth factors, may be considered an interesting tool in experimental studies of tissue regeneration. Therefore, the aim of this study was to evaluate the effects of ALT-C on oxidative stress biomarkers, histopathological alterations, VEGF production, and the degree of vascularization in the liver of traíra, *Hoplias malabaricus* (Erythrinidae), a Neotropical freshwater fish species. The three major liver functions essential for life are: (a) uptake, metabolism, storage, and redistribution of nutrients; (b) metabolism of lipophilic compounds, including xenobiotics; (c) formation and excretion of bile. All of these functions have been shown to be involved not only in physiological states, but also in diseases leading to alterations in hepatic morphology and physiology [8]. The maintenance of structure and function is relevant for the liver itself and may also ensure the integrity and homeostasis of other organs crucial for fish survival. Furthermore, the use of the fish as a model for drug-induced liver injury is promising and may support better choices taken in the early stages of drug discovery, before a compound is tested in mammals [9]. The results indicated that ALT-C improved antioxidant defenses of fish liver by decreasing the level of oxidative stress biomarkers and by increasing the activity of antioxidant enzymes. As far as we know this is the first report of such effects for a disintegrin-like/cysteine-rich protein.

## 2. Results

**Figure 1:**
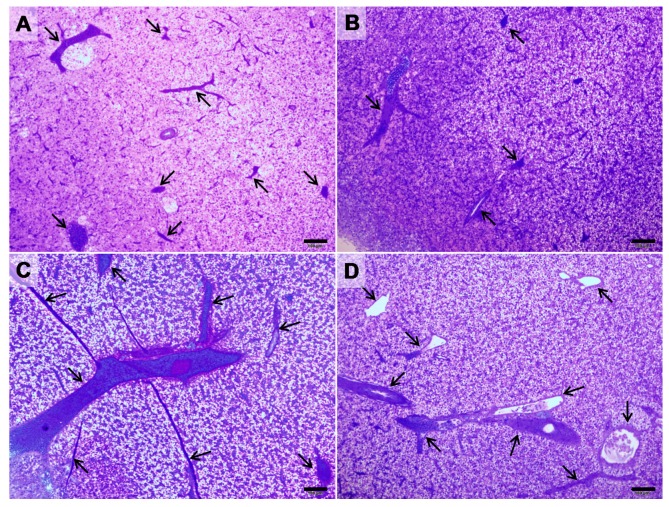
Light micrographs of sections through the liver of traíra (*H. malabaricus*) from the control group (*n* = 10, **A** and **B**) and after seven days of treatment with alternagin-C, in a single dose of 0.5 mg·kg^−1^, intra-arterial (*n* = 10, **C** and **D**). Arrows indicate blood vessels. Samples were stained with toluidine-blue/basic fuchsin. *Bar* = 100 μm.

ALT-C treatment increased the degree of liver vascularization. Figure 1 shows histological sections of the liver of fish of both experimental groups (Control and ALT-C), in which a larger number and/or size of the blood vessels present in the hepatic parenchyma of the ALT-C treated fish can be evidenced.

**Figure 2:**
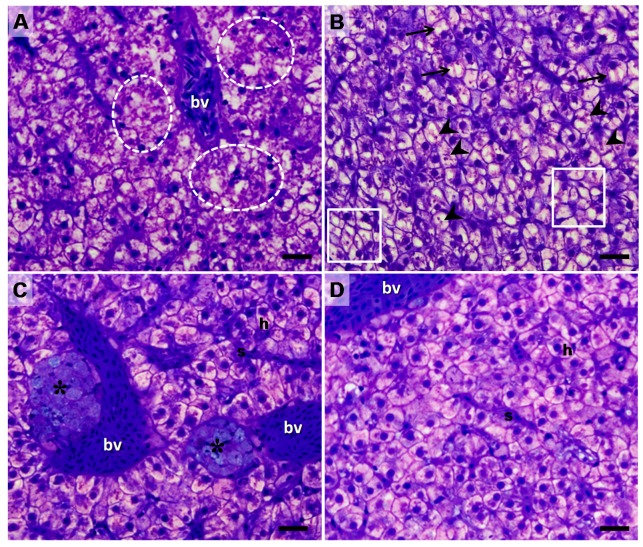
Light micrographs of sections through the liver of traíra (*H. malabaricus*) from the control group (**A**,**B**) and after seven days of treatment with alternagin-C, in a single dose of 0.5 mg·kg^−1^, intra-arterial (**C**,**D**). h: hepatocytes; s: sinusoids; bv: blood vessel; black arrow: cytoplasmic vacuoles; asterisks: melano-macrophage centers; dotted circle: cytoplasmic degeneration and architectural/structural alterations; arrow head: eosinophilic granules in cytoplasm; squar: atrophy. Samples were stained with toluidine-blue/basic fuchsin and photomicrographs were taken using 400 × magnification. *Bar* = 20 μm.

Histologically, polygonal hepatocytes with spherical and centralized nuclei clearly organized in cords surrounding sinusoid capillaries were observed in the liver of this species, characterizing the normal aspect of the tissue (Figure 2). Although the Control group exhibited normal aspect to the hepatic tissue, some structural changes were observed in some areas, such as: cytoplasmic degeneration and architectural/structural alterations, where it was not possible to see the format and the cellular delimitation, as well as the cord arrangement (Figure 2A), and cellular atrophy (Figure 2B). Other changes such as the accumulation of intracellular substances (eosinophilic-like granules, Figure 2B), the formation of cytoplasmic vacuoles (Figure 2B), and the presence of melano-macrophage centers (Figure 2C) were also observed. The ALT-C group also exhibited characteristics of normal hepatic tissue with some histopathological alterations but in lower frequencies. The liver parenchyma was homogeneous with polygonal shaped hepatocytes having a spherical nucleus and showed rare pathological features (Figure 2C,D). Few areas of morphological damages were observed like cytoplasmic degeneration in association with architectural/structural alterations. Additionally, in a smaller quantity, the melano-macrophage centers and accumulation intracellular substances were detected. Overall, the tissue of treated animals showed a smaller frequency of alterations when compared to the control group (Table 1).

**Figure 3:**
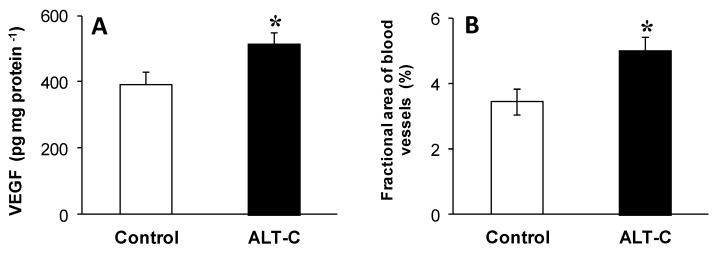
(**A**) Hepatic VEGF levels and (**B**) fractional area of the blood vessels in the liver histological sections of traíra (*H. malabaricus*) under control conditions (*n* = 10) and after seven days of treatment with alternagin-C (*n* = 10, single dose of 0.5 mg·kg^−1^, intra-arterial). Data are presented as means ± S.E.M. Asterisks indicate significant difference (*P* < 0.05) between fish groups.

The treatment with 0.5 mg·kg^−1^ of ALT-C induced hepatic angiogenesis by up-regulating the expression of VEGF. The liver tissue of fish from the ALT-C group displayed elevated (*P* < 0.05) VEGF levels (31%, Figure 3A) and a higher (*P* < 0.05) percentage of area occupied by blood vessels (1.46 fold) than the hepatic tissue of animals from the Control group (Figure 3B).

**Figure 4:**
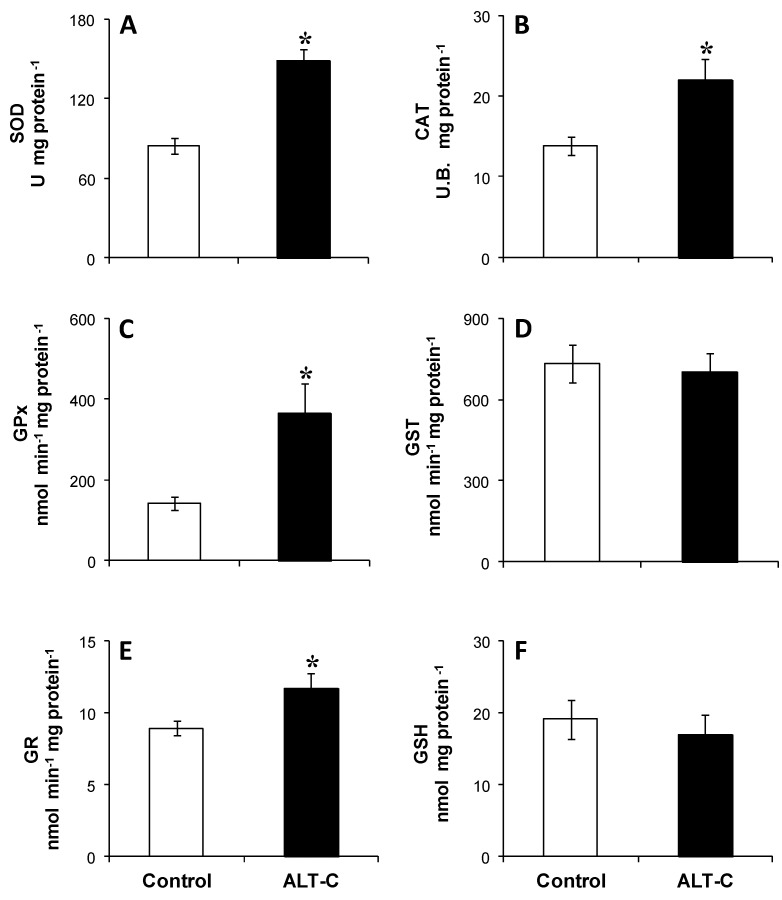
Activities of antioxidant enzymes (**A**) superoxide dismutase (SOD), (**B**) catalase (CAT), (**C**) glutathione peroxidase (GPx), (**D**) glutathione S-transferase (GST), (**E**) glutathione reductase (GR), and (**F**) reduced glutathione (GSH) levels in the liver of traíra, *H. malabaricus*, under control conditions (*n* = 10) and after seven days of treatment with alternagin-C (*n* = 10, single dose of 0.5 mg·kg^−1^, intra-arterial). Data are presented as means ± S.E.M. Asterisks indicate a significant difference (*P* < 0.05) between fish groups.

After seven days following a single-dose of ALT-C, no fish died and no changes in hepatic protein levels were observed (Control = 72. 4 ± 4.3 and ALT-C = 74.4 ± 4.1 mg·g tissue^−1^). ALT-C treatment induced significant (*P* < 0.05) increases in the hepatic superoxide dismutase (SOD), catalase (CAT), glutathione peroxidase (GPx), and glutathione reductase (GR) activities (76%, 60%, 158%, and 31%, respectively). On the other hand, glutathione S-transferase (GST) activity and reduced glutathione (GSH) content remained unaffected (Figure 4).

**Figure 5:**
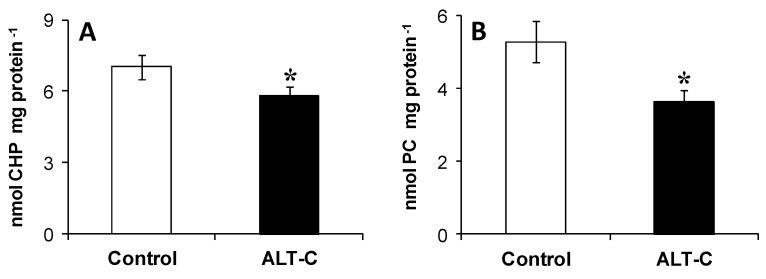
Oxidative stress indices: levels of (**A**) lipid hydroperoxide (cumene hydroperoxide—CHP—equivalents) and (**B**) protein carbonyl (PC) levels in the liver of traíra, *H. malabaricus*, under control conditions (*n* = 10) and after seven days of treatment with alternagin-C (*n* = 10, single dose of 0.5 mg·kg^−1^, intra-arterial). Data are presented as means ± S.E.M. Asterisks indicate significant difference (*P* < 0.05) between fish groups.

Additionally, the liver of fish from the ALT-C group showed significantly (*P* < 0.05) reduced levels of lipid peroxidation and protein carbonyl content (17 and 32%, respectively), when compared to the control values (Figure 5).

## 3. Discussion

This study has revealed that the administration of a single dose of ALT-C (0.5 mg·kg^−1^) improved fish hepatic tissue. When compared to controls, the liver of fish from the ALT-C group displayed higher VEGF levels and degree of vascularization, increases of the antioxidant defenses with a concomitant reduction of the oxidative damages, and a decrease in the incidence of some liver histopathological findings, usually present at a low frequency in controls, mainly cytoplasmic degeneration, hepatocyte atrophy, and architectural/structural alterations.

The fish liver is a highly vascularized organ composed of two afferent vessels, the hepatic artery and portal vein, and a single efferent vessel, the hepatic vein. The sinusoid capillaries are present among the hepatocytes and contain arterial and afferent venous blood. The treatment with ALT-C induced fish hepatic angiogenesis leading to enhanced blood flow. This process is defined as a dynamic and growth factor-dependent process leading to the formation of new blood vessels from preexisting ones and it is essential in many physiological and pathological conditions [10]. Previous studies demonstrated that ALT-C up-regulates VEGF expression and induces endothelial cell proliferation in vitro [4,6]. ALT-C competitively interacts with the *α*_2_*β*_1_ integrin, the major collagen receptor, triggering intracellular signaling typical of integrin-activated pathways and inducing the expression of several growth factors, mainly VEGF, one of the most effective angiogenic peptides [11].

Angiogenesis, the formation and maintenance of blood vessel structures, is essential for the physiological functions of tissues and VEGF plays crucial roles in the formation of new blood vessels and microvascular permeability not only in physiological, but also in pathological angiogenesis [12]. ALT-C successfully induces protein kinase B (PKB) phosphorylation, an essential signaling pathway for endothelial cell proliferation which is activated by many angiogenic factors, including VEGF [7]. ALT-C also stimulated the formation of new vessels, increased the expression of growth factors, mainly VEGF and fibroblast growth factor 1 (FGF1), and augmented the fibroblast density and collagen deposition in vivo during the healing of wounded rat skin [13,14]. VEGF stimulates endothelial cell proliferation and differentiation, increases vascular permeability, supports endothelial cell survival, adhesion, and migration, and induces endothelial cell gene expression [15].

ALT-C is also able to induce in vivo angiogenesis, in a dose-dependent way, in the wounded rat skin model after topic treatment into the wound [13]. These authors evidenced that ATL-C induced the formation of new vessels and the expression of VEGF in the injured tissue. As previously demonstrated [16], the administration of a single dose of ALT-C (0.5 mg·kg^−1^), via intra-arterial injection in *Hoplias malabaricus*, significantly increased myocardial VEGF levels after seven days. Following this line of evidence, the results of the present work pointed out the usefulness and effectiveness of ALT-C, as a pro-angiogenic desintegrin-like protein, by increasing the hepatic VEGF levels and the area occupied by blood vessels, after intra-arterial administration in fish, an alternative animal model. The use of fish in scientific research is growing due to both the expansion of the fish farming industry and an emergent awareness of questions concerning the humane use of mammalian models in basic research and chemical testing [17].

The changes that ALT-C induced in the hepatic microcirculation could result in a better oxygen and fuel supply to the fish liver. Oxygen supply is crucial to cell metabolism and reactive oxygen species (ROS) production mostly depends on appropriated oxygen levels [18,19]. ROS generation is a physiological process due to the oxidative metabolism of the cell or the activity of specific enzymatic complexes. Both the enzymatic and non-enzymatic antioxidant system are essential for the cellular response in order to deal with oxidative stress under a physiological condition [20]. Oxidative stress occurs when the critical balance between oxidants and antioxidants is disrupted due to the depletion of antioxidants or excessive accumulation of the ROS, or both [21,22], and can cause damage to lipids, protein, and DNA, leading to a disruption of cellular function and tissue injury [23].

In the present study, the enhanced hepatic fish tissue blood flow could have stimulated ROS production, such as the superoxide anion (O_2_^•−^), hydrogen peroxide (H_2_O_2_), and hydroxyl radical (HO^•^). Increased ROS in cells may lead to an elevation of antioxidant enzymes as an adaptive response to neutralize the harmful effects of free radicals in the liver tissue. SOD and CAT enzymes have connected functions since SOD catalyses the conversion of the superoxide anion radical to H_2_O and H_2_O_2_, which is detoxified by both CAT and GPx activity. The SOD-CAT system provides the first defense line against oxygen toxicity [24] and is usually used as a biomarker of ROS production [25,26]. The increased SOD and CAT activities induced by ALT-C in the liver of traíra indicate an elevated antioxidant status attempting to neutralize the impact of the ROS and, consequently, contributing to the reduction of basal protein and lipid oxidation levels. ROS are generated in a wide range of normal physiological conditions, resulting in basal levels of lipid and protein oxidation. When the cellular production of ROS overwhelms its antioxidant capacity, damage to cellular macromolecules such as lipids, proteins, and DNA above basal levels may occur, characterizing oxidative stress.

Traíras treated with ALT-C also displayed significant increases in the hepatic GPx and GR activities, while the GST activity remained unchanged. GPx protects tissues from oxidative damage by reducing H_2_O_2_ and a wide range of organic hydroperoxides that form an important group of toxic compounds produced by oxygen metabolism, therefore preventing lipid peroxidation in the membranes, and acts as a ROS scavenger [27,28]. The higher increase in the hepatic GPx activity in fish treated with ALT-C was probably enough to efficiently detoxify the H_2_O_2_ and the lipid hydroperoxides, leading to lower lipid peroxidation levels. The major detoxification function of GPx is the termination of radical chain propagation by quick reduction to yield ROS [29].

The detoxification of ROS and hydroperoxides by GPx involves a concomitant oxidation of reduced GSH to its oxidized form (GSSG). This GSSG is then reduced to GSH by GR at the expense of nicotinamide adenine dinucleotide phosphate (NADPH), which is recycled by the pentose phosphate pathway [30]. To maintain suitable GSH levels to the GPx and GST activities, a higher GR activity was detected in the fish of the ALT-C group when compared to the control group. GR plays a critical role in maintaining the larger glutathione pool in the reduced form, while GSH is the dominant non-protein thiol and is essential for protecting cells from oxidative damage and the toxicity of xenobiotic electrophiles, as well as maintaining redox homeostasis [31].

GSTs constitute a large multigene family of phase II detoxification enzymes involved in the conjugation of glutathione (GSH) to electrophilic compounds, such as xenobiotics, through thioether linkages, leading to the formation of conjugates that are more readily excreted and typically less toxic [32,33]. No differences were observed between experimental groups regarding hepatic GST activity indicating that ALT-C did not activate the liver detoxification pathways. These results reinforce the hypothesis that ALT-C, even though a disintegrin-like protein extracted from the snake venom, can be an important therapeutic tool. The liver is particularly susceptible to chemical injury because of its strategic location, prominent blood supply and prevalent role in the biotransformation of xenobiotics [34]. The formation of electrophilic reactive metabolites is considered to be an undesirable trait of drug candidates on the grounds of evidence linking this liability with drug-drug interactions, end-organ toxicity, and genotoxicity [35].

It is worth pointing out that the VEGF can induce Nrf2 (nuclear factor erythroid-2-related factor 2) expression by the activation of downstream effectors, including the protein kinase C (PKC), serine/threonine-protein kinase (Raf), and extracellular signal-regulated kinase (ERK1/2)/phosphatidylinositol 3-kinase (PI3K)-focal adhesion kinase pathways [36]. Nrf2 is a redox-sensitive transcription factor that binds to the antioxidant response element (ARE), leading to an upregulation of antioxidant gene expression that controls the elimination of ROS and electrophiles [37]. The results from this study indicate a possible activation of VEGF-Nrf2 signaling by ALT-C.

Two of the more obvious differences between fish and mammals are that fish have a lower perfusion rate and 50-fold slower bile flow rate [38,39], suggesting that the liver tissues of fish are more susceptible to damage by chemical agents. Due to these anatomic and physiologic considerations, histological changes often occur in the liver from control fish. The morphologic features of liver toxicity are often exacerbations of findings that may be observed in normal or control fish [38]. In the present investigation, the induction of hepatic angiogenesis and antioxidant defense systems by ALT-C with concomitant reductions in the basal oxidative damages was able to lead to an improvement of the major liver histological features. Future research directions may also be highlighted.

## 4. Conclusions

In summary, ALT-C treatment offered a considerable beneficial effect on fish liver vascularization supporting in vivo pro-angiogenic activity of this desintegrin-like peptide extracted from snake venom. The results point out that ALT-C induces angiogenesis through increased VEGF levels producing better hepatic tissue morphology and indicating the potential application of ALT-C in therapies for tissue repair. Furthermore, these ALT-C-induced changes in the hepatic tissue improved antioxidant defense systems. However, the action of ALT-C on different hepatic cell populations and the specific signaling pathways involved in its effects remains to be elucidated in future studies, as well as to validate the translation of the model to humans.

## 5. Materials and Methods

This study was performed under the approval of the Animal Ethics Committee of the Federal University of São Carlos (CEUA/UFSCar—Approval #049/14 September 2012) and in accordance with the Guide for Care and Use of Laboratory Animals published by National Institutes of Health and the ethical guidelines.

### 5.1. Animals

Adult specimens of *Hoplias malabaricus* (traíra, 131.3 ± 5.4 g, mean ± E.P.M.) were obtained from the Santa Candida Fish Farm (Santa Cruz da Conceição, São Paulo, Brazil). Fish were acclimated for 60 days prior to experimentation in 500 L holding tanks equipped with a continuous supply of well-aerated (*P_w_*O_2_ > 130 mmHg) and dechlorinated water at a constant temperature (24 °C) under a natural photoperiod (~12 h:12 h). During this period, fish were fed weekly with small live fishes.

### 5.2. Alternagin-C (ALT-C)

ALT-C was isolated from *Rhinocerophis alternatus* lyophilized venom (provided by the Institute Butantan, São Paulo, Brazil) by two steps of gel filtration followed by anion exchange chromatography. ALT-C is purified as a precursor peptide with a metalloprotease domain, from which it is released after proteolytic processing, resulting in a form with disintegrin and cysteine-rich domains according to the procedures previously described [11].

### 5.3. Experimental Design and Treatment

Fish were divided into two groups (*n* = 10 in each group): one group received a single 0.5 mg·kg^−1^ intra-arterial injection of alternagin-C (ALT-C group), and the other group was treated with sterile saline (Control group). Fish were anaesthetized in a 0.1% (*w*/*v*) solution of benzocaine and then placed onto an operating table. An indwelling cannula was implanted into the third afferent branchial artery on the left side using polyethylene tubing (PE 10) allowing drug injection according to the procedures previously described [40]. This cannula was filled with saline and heparin solution (NaCl 0.9%, 100 IU·mL^−1^ of heparin) and used to deliver injections of 0.2 mL of sterile saline or ALT-C (0.5 mg·kg^−1^). To ensure complete drug delivery, after each injection, the cannula was cleaned with a new solution of saline (0.1 mL) and sutured in place. The chosen dose of ALT-C was based on our previous study [16]. After seven days, fish were euthanized through cerebral concussion followed by anterior spinal cord section. The fish liver was dissected and samples were either fixed in 4% paraformaldehyde (PFA) in 0.1 M phosphate buffer pH 7.2 for morphological analyses or frozen into liquid nitrogen and stored at −80 °C until an analysis of the VEGF levels or stress oxidative biomarkers.

### 5.4. Liver Morphological Analysis

For histomorphology, liver samples fixed in buffered PFA were dehydrated in ethanol crescent series, embedded in historesin (Leica, Wetzlar, Germany), and the sections (3 μm thickness) were stained with Toluidine blue and basic fuchsin. The slides were analyzed using an Olympus BX51 light microscope (Olympus, Ballerup, Denmark) equipped with a camera connected to a computer using Olympus DP2-BSW software (Version 2.2, Olympus, Ballerup, Denmark, 2008). For the quantification of liver vascularization, the total area occupied by the histological section and the total area occupied by the blood vessels (arteries and veins) were measured with the help of the image analysis software (Motic Image Plus 2.0, Motic China Group Co., Ltd., Hong Kong, China, 2006). The fractional area of the blood vessels was calculated through the ratio between the total area occupied by these vessels and the total area occupied by the histological section (six-eight non-contiguous fields/section/fish), multiplied by 100. The mean values for each fish were calculated and these values were used to determine the average of each experimental condition. The presence of histological alterations was also evaluated by a randomized blind method.

### 5.5. Quantification of VEGF

Frozen livers were homogenized in RIPA buffer (10 mM Tris pH 7.4, 100 mM NaCl, 1 mM EDTA, 1 mM EGTA, 1% Triton X-100, 10% glycerol, 0.1% SDS, 0.5% Na deoxycholate, and protease inhibitor cocktail from Sigma—P8340) and centrifuged at 10,000 rpm for 40 min at 4 °C. VEGF levels were quantified using the Murine VEGF Mini ELISA Development kit (cat. no. 900-M99, Peprotech, Rocky Hill, NJ, USA), according to the manufacturer’s instructions. Briefly, 100 uL of standards (recombinant murine VEGF) or liver homogenates were added in triplicate on each well, previously coated overnight with polyclonal rabbit anti-murine VEGF capture antibody. After 2 h incubation, wells were washed and incubated with biotinylated rabbit anti-murine VEGF and avidin-HRP conjugate. Following another wash, ABTS (2,2′-Azino-bis 3-ethylbenzothiazoline-6-sulfonic acid) substrate solution was added to wells and color developed in proportion to the amount of VEGF bound in the initial step. The plate was read on a Spectra Max M5 plate reader (Molecular Devices, LLC, Sunnyvale, CA, USA) with an absorbance of 405 nm. Data are expressed as the VEGF content in pg of mg protein^−1^.

### 5.6. Biomarkers for Antioxidant Defense and Oxidative Damage

#### 5.6.1. Antioxidant Defenses

Samples of frozen tissue were homogenized in 0.1 M Na^+^/K^+^ phosphate buffer pH 7.0 at 18,000 rpm. Homogenates were centrifuged at 14,000 rpm for 30 min at 4 °C. The supernatants were used for the oxidative stress biomarker assays described below.

Superoxide dismutase (SOD) activity was evaluated based on the determination of the cytochrome *c* reduction rate by superoxide anions, monitored at 550 nm [41]. The reaction mixture (1 mL) contained 50 mM Na^+^/K^+^ phosphate buffer (pH 7.8), 0.1 mM EDTA, 1 mM xanthine, 20 mM cytochrome *c*, tissue homogenates, and a sufficient amount of xanthine oxidase to produce a rate of cytochrome *c* reduction of 0.025 absorbance units/min. One unit of SOD activity was calculated as the amount of enzyme causing 50% of the maximum inhibition of the cytochrome *c* reduction.

Catalase (CAT) activity was measured as the decrease of H_2_O_2_ concentration at 240 nm [42]. Decays in absorbance were recorded for 15 s in 50 mM sodium phosphate buffer (pH 7.0) containing 15 mM H_2_O_2_ and tissue homogenates. CAT, together with glutathione peroxidase, acts by removing the hydrogen peroxide. However, CAT is responsible for the detoxification of elevated levels of H_2_O_2_ (as the concentration contained in the reaction mixture). The non-enzymatic oxidation of H_2_O_2_, obtained using water instead of enzyme samples, was subtracted from tissue sample decay rates. CAT activity was expressed as Bergmeyer units (B.U.) per mg of protein [43].

Glutathione peroxidase (GPx) activity was assessed by a coupled assay with glutathione reductase (GR)-catalyzed oxidation of NADPH [44]. The consumption of NADPH was recorded at 340 nm in media containing 50 mM sodium phosphate buffer (pH 7.0), 1 mM EDTA, 0.2 mM NADPH, 1 mM sodium azide, 1 U·mL^−1^ GR, 1 mM GSH, 0.2 mM H_2_O_2_, and tissue homogenates. Sodium azide was used to block catalase activity. The non-enzymatic oxidation of GSH was determined by using water instead of the enzyme fraction, and its reaction rate was subtracted from the rates of liver homogenates in order to determine the true enzymatic activity. The activity of GPx was expressed as mU mg protein^−1^ and 1 mU was defined as 1 nmol of NADPH consumed min^−1^·mL^−1^ of the sample, using NADPH molar extinction coefficient (*ε*_340_ = 6.2 mM^−1^·cm^−1^).

Glutathione-S-transferase (GST) activity was evaluated using 1-chloro-2, 4-dinitrobenzene (CDNB) as a substrate [45]. The assay mixture contained 1 mM CDNB in ethanol, 1mM GSH, 100 mM potassium phosphate buffer (pH 7.0), and tissue homogenates. The formation of adduct S-2, 4-dinitrophenyl glutathione was monitored by the increase in absorbance at 340 nm against a blank. The activity was measured as the amount of enzyme catalyzing the formation of 1 nmol of the product min^−1^·mg^−1^ of protein, using the CDNB-GSH conjugate molar extinction coefficient (*ε*_340_ = 9.6 mM^−1^·cm^−1^).

Glutathione reductase (GR) activity was measured at 340 nm [46]. The reaction system of 1 mL contained: 1 mM oxidized glutathione (GSSG), 0.2 mM NADPH, 0.5 mM EDTA 50 mM K^+^ phosphate buffer (pH 7.0), and a suitable amount of the glutathione reductase sample to change the absorbance from 0.05 to 0.30 per min at 340 nm. The GR activity in the samples was calculated by using the extinction coefficient of NADPH. The net rate for each sample was obtained by subtracting the rate obtained for the blank (the blank measures the spontaneous oxidation of NADPH). Under these conditions, the oxidation of 1 µmol of NADPH min^−1^·mg^−1^ of protein was used as a unit of GR activity.

The estimate of reduced glutathione (GSH) content was analyzed according to using Elmann’s reagent (DTNB) [47]. Supernatants of the acid extracts (1:1 *v*/*v* with 12% TCA) were added to 0.25 mM DTNB in a 0.1 sodium phosphate buffer pH 8.0, and a thiolate anion formation was determined at 412 nm against a GSH standard-curve. DTNB-reactive thiols levels (as GSH equivalents) were expressed as nmol·mg protein^−1^.

#### 5.6.2. Oxidative Damage

Lipid peroxidation (LPO) was determined by a FOX (ferrous oxidation-xylenol orange) assay for lipid hydroperoxide [48]. The principle of the FOX method is based on the oxidation of ferrous ions to ferric by the hydroperoxide activity under acidic conditions. Liver samples were homogenized in 0.1 M Na^+^/K^+^ phosphate buffer pH 7.0 at 18,000 rpm. Homogenates were centrifuged at 14,000 rpm for 30 min at 4 °C. The lipid hydroperoxide (LHP) was determined with 100 μL of supernatant samples (previously treated with 10% trichloroacetic acid—TCA) and 900 μL of reaction mixture containing 0.25 mM ammonium ferrous sulfate, 25 mM H_2_SO_4_, 0.1 mM xylenol orange, and 4 mM butylated hydroxytoluene in 90% (*v*/*v*) methanol. Blanks contained all components without supernatant. Mixtures were incubated for 30 min at room temperature prior to measurements at 560 nm. LHP levels were quantified using a calibration curve obtained with cumene hydroperoxide (CHP—2 to 200 nmol) in the corresponding reaction medium. Data are normalized by the amount of CHP equivalents per mg protein in the liver homogenate.

Protein carbonyl (PC) content was determined by colorimetric 2,4-dinitrophenylhydrazine (DNPH) [49]. Liver samples were homogenized in 50 mM K_2_PO_4_ buffer containing 1 mM EDTA and 40 μg·mL^−1^ phenylmethylsulfonyl fluoride (PMSF) and then centrifuged at 10,000 rpm for 10 min. Supernatant samples were incubated with 10 mM DNPH in 2.5 M hydrochloric acid at room temperature for 1 h, in a dark environment with a 15 min interval of vortexing. After that, these reactive mixtures were precipitated with 50% TCA and centrifuged for 10 min at 10,000 rpm. The pellets were washed three times with 1 mL of ethanol/ethyl acetate (1:1 *v*/*v*) mixture and dissolved in 6 M guanidine hydrochloride. The carbonyl content was measured spectrophotometrically at 370 nm. Carbonyl content was calculated using the molar absorption coefficient of 22,000 M^−1^·cm^−1^ relative to protein concentration.

### 5.7. Total Protein

The concentration of total protein in liver homogenates was carried out with Coomassie Brilliant Blue G-250 [50] adapted to a microplate reader [51] using bovine albumin as a standard.

### 5.8. Statistical Analysis

Data are shown as means ± SEM. Biochemical and morphometric data of the two experimental groups (Control and ALT-C) were compared and analyzed using a Student *t*-test or the non-parametric Mann-Whitney test, respectively. All tests were performed using GraphPad Prism software (Version 5.00, GraphPad Software, Inc., San Diego, CA, USA, 2007) and the data significance was designated at *P* < 0.05.

## Figures and Tables

**Table 1:** Liver histopathology of *H. malabaricus* after seven days of treatment with alternagin-C (single dose of 0.5 mg·kg^−1^, intra-arterial).

Lesion	Control	ALT-C
Aneurism/Haemorrhage/Hyperaemia	A	A
Architectural and structural alterations	+	0+
Atrophy	+	A
Cytoplasmic vacuoles	0+	0+
Cytoplasmic degeneration	++	+
Eosinophilic granules in cytoplasm	0+	0+
Hypertrophy	A	A
Melano-macrophages centers	+	0+
Necrosis	A	A
Nuclear alterations	A	A

A: absent; 0+: very low frequency; +: low frequency; ++: frequent.
